# *In silico* and *in vivo* investigations using an endocavitary photoplethysmography sensor for tissue viability monitoring

**DOI:** 10.1117/1.JBO.25.2.027001

**Published:** 2020-02-28

**Authors:** Subhasri Chatterjee, Zaibaa Patel, Mohamed A. Thaha, Panayiotis A. Kyriacou

**Affiliations:** a City, University of London, Research Centre for Biomedical Engineering, London, United Kingdom; b Queen Mary, University of London, National Bowel Research Centre, Blizard Institute, Barts and the London School of Medicine and Dentistry, London, United Kingdom; c The Royal London Hospital, Barts Health NHS Trust, Department of Colorectal Surgery, London, United Kingdom

**Keywords:** photoplethysmography, Monte Carlo, endocavitary sensor, signal-to-noise ratio, penetration depth

## Abstract

**Significance:** Colorectal cancer is one of the major causes of cancer-related deaths worldwide. Surgical removal of the cancerous growth is the primary treatment for this disease. A colorectal cancer surgery, however, is often unsuccessful due to the anastomotic failure that may occur following the surgical incision. Prevention of an anastomotic failure requires continuous monitoring of intestinal tissue viability during and after colorectal surgery. To date, no clinical technology exists for the dynamic and continuous monitoring of the intestinal perfusion.

**Aim:** A dual-wavelength indwelling bowel photoplethysmography (PPG) sensor for the continuous monitoring of intestinal viability was proposed and characterized through a set of *in silico* and *in vivo* investigations.

**Approach:** The *in silico* investigation was based on a Monte Carlo model that was executed to quantify the variables such as penetration depth and detected intensity with respect to the sensor–tissue separations and tissue perfusion. Utilizing the simulated information, an indwelling reflectance PPG sensor was designed and tested on 20 healthy volunteers. Two sets of *in vivo* studies were performed using the driving current intensities 20 and 40 mA for a comparative analysis, using buccal tissue as a proxy tissue-site.

**Results:** Both simulated and experimental results showed the efficacy of the sensor to acquire good signals through the “contact” to a “noncontact” separation of 5 mm. A very slow wavelength-dependent variation was shown in the detected intensity at the normal and hypoxic states of the tissue, whereas a decay in the intensity was found with the increasing submucosal-blood volume. The simulated detected-to-incident-photon-ratio and the experimental signal-to-noise ratio exhibited strong positive correlations, with the Pearson product-moment correlation coefficient R ranging between 0.65 and 0.87.

**Conclusions:** The detailed feasibility analysis presented will lead to clinical trials utilizing the proposed sensor.

## 1 Introduction

Colorectal cancer is one of the leading causes of mortality and morbidity in the world.^1^ It is the third most common malignancy and the fourth leading cause of cancer-related deaths worldwide.^2^ Surgery is the primary treatment for most colorectal cancers, which often involves excision of the tumor-bearing intestine followed by the formation of an intestinal anastomosis to restore intestinal continuity. Anastomotic failure occurs in 2% to 10% of patients, mainly due to inadequate vascularity surrounding the anastomosis.^3^ Monitoring of intestinal perfusion during and after surgery is extremely important for improving the surgical outcome. However, there are no reliable tools available to directly monitor intestinal perfusion across a newly constructed anastomosis and hence, the prediction of anastomotic failure remains a clinical challenge.

The intraoperative assessment of intestinal viability is currently based on visual inspection of the color, arterial pulsation, and presence of peristalsis.^4^ There are several techniques that could be used for monitoring intestinal tissue perfusion, such as endoscopic fluorescence imaging, laser Doppler flowmetry, and narrow band imaging.^5^^–^^7^ Such techniques are primarily confined to research tools and have not been adopted for mainstream clinical practice due to the incapability of allowing continuous and dynamic monitoring of intestinal viability and the inability to transition between intraoperative and postoperative monitoring.

Optical techniques are known to be used for continuous hemodynamic monitoring of tissue without inducing perturbation in the tissue environment. Photoplethysmography (PPG) is an optical technique used for the detection of the changes in blood volume in a vascular tissue-bed.^8^ By extracting the “ac” and “dc” components from a PPG signal acquired from a perfused tissue-volume, the information regarding the pulsatile (i.e., arterial blood) and nonpulsatile (i.e., bloodless tissue, venous, and capillary blood) compartments of the tissue can be interpreted. PPG is widely known for its application in pulse oximetry for the measurement of arterial oxygen saturation (${\mathrm{SpO}}_{2}$).^9^ Recently, there has been a plethora of interest in extending the application of PPG beyond pulse oximetry, for example, in the assessment of vascular mechanics, heart rate, blood pressure, tissue perfusion, respiration, and mental stress.^10^^–^^16^

To continuously monitor the intestinal perfusion and oxygenation, an endocavitary PPG sensor has been proposed.^17^ The proposed sensor can be inserted through the anal orifice of a patient to capture the PPG signal by illuminating the colon utilizing light emitters of dual wavelength, corresponding to red and infrared. The reflected light may be detected from the inner wall of the intestine and analyzed to extract the physiological information based on the tissue perfusion and oxygenation.

An optical sensor in contact with the tissue surface allows the incident optical illumination to directly pass through the tissue volume, whereas a separation between them in a noncontact geometry compels the photons to travel a certain distance through the air before reaching the tissue bed. This may result in a difference in the optical quantities (e.g., depth of penetration, detected intensity, optical path, etc.) between the contact and noncontact geometries. In an endocavitary environment where a sensor is inserted within a hollow intestinal tube, it is very difficult to predict the exact location and orientation of the sensor. Therefore, prior to applying the proposed sensor clinically, it is of utmost importance to evaluate the dependence of the optical quantities on the sensor–tissue configuration pertinent to the reflectance mode PPG monitoring from intestine. The Monte Carlo method is the most convenient approach to model the optical quantities in a certain sensor–tissue configuration.^18^ In this paper, a rigorous analysis is presented to evaluate the efficacy of the sensor using a robust Monte Carlo model that has been evaluated against a set of *in vivo* investigations. The combined knowledge obtained through the simulations and experiments presented in this paper will inform the design and application-protocols of the proposed sensor, leading to the future development of a sensing technology for the continuous and dynamic intestinal viability monitoring.

## 2 Methodology

**Fig. 1:**
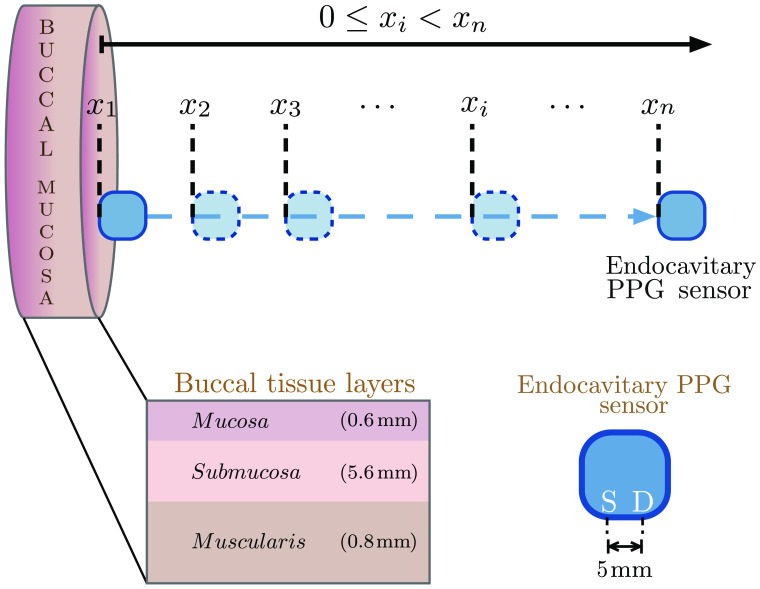
*In silico* experiment setup is illustrated. The endocavitary sensor is placed at several distances, $x_{i}$, from the buccal tissue. Concerning the surface of the buccal tissue, let $x_{1}$ be 0 mm and $x_{n}$ be 10 mm. Illustrations show the stratification of buccal tissue layers and the geometry of the reflectance mode PPG sensor.

The proposed endocavitary PPG sensor operates in a reflectance geometry that comprises the optical sources illuminating light at wavelengths 660 nm (red) and 880 nm (infrared), and a detector at a distance of 5 mm from the source.^17^ In this paper, the designed sensor was characterized by the steps of an *in silico* simulation followed by an *in vivo* investigation. Due to the inconvenience to physically access the colon tissue for controlled experiments, buccal tissue was adopted as a surrogate, as the tissue shares anatomical and physiological similarities with the colon tissue.^19^ Initially, a heterogeneous Monte Carlo model of buccal tissue volume was developed and exposed to the sensor geometry to quantify the light–tissue interaction variables. The model was explored at various states of tissue perfusion and oxygen saturation, replicating several possible states of intestinal perfusion. The experimental work involved the acquisition of the PPG signals from 20 healthy volunteers using the designed sensor followed by an analysis of the signals. Both studies followed the same evaluation where the sensor–tissue separation was adjusted to the contact to noncontact configurations as shown in Fig. 1.

### 2.1 *In Silico* Characterization

A MATLAB (Mathworks, Inc.) platform was chosen for the algorithm development and a multithread programming environment was used for facilitating the simulation. A 64-bit operating system with an installed memory of 24 GB and an Intel Xenon CPU (2.40 GHz, 2 processors) was dedicated for the simulation.

#### 2.1.1 Monte Carlo methodology

A three-dimensional (3-D) heterogeneous model of buccal tissue was characterized by its wavelength-dependent optical properties, such as scattering coefficient $\mu_{s}$, absorption coefficient $\mu_{a}$, and scattering anisotropy g. The virtual photon packets were launched to the tissue with an initial weight of w=1. Photons were propagated through the medium by step sizes (l), determined as the function of the total interaction coefficient $\mu_{t}=\mu_{a}+\mu_{s}$, i.e.,

$$l=-\frac{\ln(\xi )}{\mu_{t}},$$ (1)

where ξ is a computer-generated pseudorandom number (0<ξ<1). The corrections due to the reflection and refraction at the tissue boundary and the interfaces between two tissue layers were taken into consideration. Absorption of the photon packet was simulated at each interaction site by deduction of a fraction of the photon weight (i.e., $\Delta w=\frac{\mu_{a}}{\mu_{t}}.w$). Scattering was simulated by calculating the direction cosines after scattering through an angle θ, calculated using the Henyey–Greenstein phase function whereas the azimuth was randomly generated between 0 and 2π^20^

$$\theta =\cos^{-1}\;\frac{1}{2g}[1+g^{2}-{\left(\frac{1-g^{2}}{1-g+2g\xi }\right)}^{2}]\;\phi =2\pi \xi .$$ (2)

The steps were repeated until the photon packet was detected or discarded. The process repeated until the desired accuracy was obtained. The accuracy of the Monte Carlo simulation is quantified by the “convergence rate,” the lower the convergence rate, the higher the accuracy. For a low convergence rate, a very high number of photons ($≃10^{8}$) were simulated. Further details of the Monte Carlo modeling strategy are discussed in our earlier publication.^18^

#### 2.1.2 Tissue description

A buccal tissue model consisting of three layers was used in this work as shown in Fig. 1, where the mucosa was the inner most layer that progresses toward the outer muscularis surface. The optical and anatomical properties for the buccal layers were adopted from published literature.^21^^–^^24^ The “perfusion change” in the tissue was simulated as a change in blood volume in the submucosal layer. In order to consider the volumetric contribution of the absorbers, the individual absorption properties of blood and tissue were considered in the submucosal absorption properties, with the calculated absorption coefficient $\mu_{a_{sm}}$ as

$$\mu_{a_{sm}}=(1-V)\mu_{a_{sm_{\text{baseline}}}}+V[{\mathrm{StO}}_{2}\mu_{a_{\mathrm{HbO}}}+(1-{\mathrm{StO}}_{2})\mu_{a_{\mathrm{Hb}}}],$$ (3)

where $\mu_{a_{\mathrm{Hb}}}$ and $\mu_{a_{{\mathrm{HbO}}_{2}}}$ are the absorption coefficients of deoxyhemoglobin and oxyhemoglobin, respectively, and V and ${\mathrm{StO}}_{2}$ are the volume and total oxygen saturation of blood present in the submucosa layer, respectively. The optical properties of the individual tissue layers were calculated using the baseline absorption coefficient of the bloodless tissue and the optical properties of oxy- and deoxyhemoglobin. The optical properties for blood having a hematocrit (Hct, proportion of red blood cells in total blood volume) of 45% were collected from published literature.^25^ The calculation of the arterial and venous blood absorption coefficients has been discussed in detail in our previous publication.^18^ The details of the parameters used in the simulation are illustrated in Table 1: volume fraction of total blood in each tissue-layer (V) and the optical properties (i.e., absorption coefficient, scattering coefficient, anisotropy factor, and refractive index). The consideration for a volumetric absorbance distribution leads to a proportional increase or decrease in the baseline tissue volume associated with a decrease or increase in the blood volume.

**Table 1:** The parameters of the buccal tissue model: optical properties at red (660 nm) and infrared (880 nm) wavelengths and blood volume. The optical properties include the coefficients for absorption ($\mu_{a}$) and scattering ($\mu_{s}$), the scattering anisotropy (g), and the refractive index (R.I.). Wavelength-dependent optical parameters are written in the form: $\frac{660\;\mathrm{nm}}{880\;\mathrm{nm}}$.

Buccal layers	$\mu_{a}$ (${\mathrm{mm}}^{-1}$)	$\mu_{s}$ (${\mathrm{mm}}^{-1}$)	g	R.I.	V
Mucosa	$\frac{0.026}{0.026}$	$\frac{26.33}{22.05}$	$\frac{0.9}{0.9}$	1.4	0.08
Submucosa	$\frac{0.039}{0.05}$	$\frac{9.17}{8.249}$	$\frac{0.939}{0.956}$	1.36	0.04
Muscle	$\frac{0.28}{0.18}$	$\frac{9.499}{9.157}$	$\frac{0.914}{0.928}$	1.357	0.1

#### 2.1.3 Execution of the model

The *in silico* model replicates a reflectance mode dual-wavelength sensor that is capable of working in both contact and noncontact geometries. A fixed source–detector separation of 5 mm was used for all simulations. A Gaussian beam of a beam diameter of 1 mm was simulated. The radial position r of the beam was randomly sampled from the following probability distribution function:

$$p(r)=\frac{e^{\frac{-r^{2}}{b^{2}}}2\pi r}{\pi b^{2}},$$ (4)

where b is the $1/e^{2}$ radius (i.e., the radius where the intensity values fall to $1/e^{2}$ of its axial values). Photons were emitted from the source in a direction perpendicular to the tissue surface. The detector was defined to have an active area of $0.66\;{\mathrm{mm}}^{2}$, similar to the designed sensor.

In the tissue model, the arterioles present in the submocusa supposedly contribute to the pulsatile component of the PPG, whereas the submucosal venules, mucosa (containing the capillary network), and muscle contribute to the nonpulsatile component of PPG.^26^ The arteriovenous blood volume ratio in the submucosa was 1:5.^27^ In order to investigate the sensor-performance at different tissue physiologies, various states of tissue perfusion and oxygenation were simulated in a contact sensor–tissue reflectance geometry. The total submucosal blood volume was incremented from 2% to 10% through a step of 2% (in Table 1, the nominal submucosal blood volume of 4% is mentioned).^23^ For each blood volume, simulations were run for the arterial oxygen saturations from 50% to 100% through a step of 5%. Therefore, 55 sets of simulations were carried out for each wavelength, that is, 110 sets of simulations in total, each detecting $10^{8}\text{ photons}$.

In order to investigate the sensor performance from the contact through the noncontact geometries, simulations were carried out for a range of sensor–tissue separation (0 to 10 mm), considering the submucosal tissue perfusion in its nominal form (i.e., total blood volume 4% and oxygen saturation 95%). A $10^{8}$ number of photons were detected at each wavelength and the corresponding intensities and penetration depths were calculated.

The main quantifiable parameters in the simulation were “detected intensity” and “penetration depth.” The “detected intensity” (I) was simulated as the mean weight of all detected photons following

$$I=\frac{1}{N_{\det}}\overset{i=N_{\det}}{\underset{i=1}{\sum }}w_{\det_{i}},$$ (5)

where $N_{\det}$ is the number of detected photons and $w_{\det_{i}}$ is the weight of the i’th detected photon. The “depth of penetration” (D) was determined as the mean of the maximum z coordinates of all detected photons.

To account for the photon loss (i.e., the photons that have been terminated without being detected), we denoted a parameter named “detected-to-incident photon ratio” (DIR) as the ratio of the numbers of detected to the incident photons:

$$\mathrm{DIR}=\frac{N_{\det}}{N_{\mathrm{inc}}},$$ (6)

where $N_{\mathrm{inc}}$ is the number of total incident (including detected and discarded) photons.

### 2.2 *In Vivo* Investigation

An *in vivo* investigation was performed on 20 healthy participants, where the developed endocavitary sensor was placed within the oral cavity. Similar to the *in silico* study and the schematic presented in Fig. 1, the effects of sensor–tissue separation on the PPG signals from the buccal tissue were evaluated.

#### 2.2.1 Sensor design

**Fig. 2:**
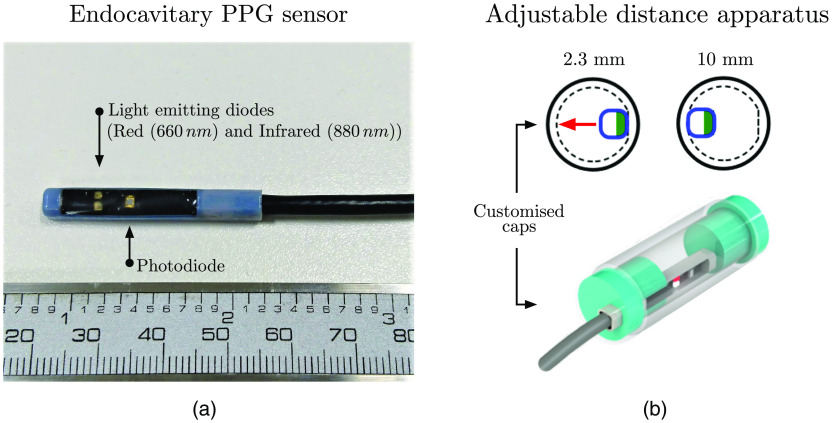
Demonstration of the developed sensor and the *in vivo* set-up: (a) the endocavitary sensor and (b) the adjustable distance apparatus with examples of mechanical drawings of customized caps at two distances, 2.3 and 10 mm, and a 3-D visualization of the full assembly for measurements at 2.3 mm. The inner circle (dashed) of the customized caps corresponds to the measurement distance of 2.3 mm, where the flat indent indicates the sensor direction. By adjusting the sensor toward the left, away from the flat indent as indicated with the red arrow, the separation distance between the sensor and buccal mucosa increases to 10 mm.

The endocavitary PPG sensor, Fig. 2(a), consists of a printed circuit board (PCB), encapsulated by a 3-D-printed capsular casing, with the overall dimensions of $36\times 6\;{\mathrm{mm}}^{2}$ (l×h). The PCB consists of a red and infrared surface mount light-emitting diode (LED), with peak wavelength emissions of 660 (KP-2012SRC, Kingbright, Taiwan) and 880 nm (KP-2012SF4C, Kingbright, Taiwan), respectively. For the detection of the reflected light from the tissue, a flat-top photodiode with an active area of $0.65\;{\mathrm{mm}}^{2}$ and peak wavelength sensitivity of 900 nm (SR10BP, Excelitas Technologies, Massachusetts) was used. The separation distance from the center of the photodiode to the center of each LED was 5 mm. To adjust the driving current of the LEDs, which proportionally adjusts the light intensities, and to condition the acquired PPG signals, a PPG processing system, ZenPPG, which was developed by the Research Centre for Biomedical Engineering at City, University of London, was utilized.^28^ The portable, battery-operated ZenPPG permits the acquisition of raw, dual-wavelength PPG signals prior to the analogue-to-digital conversion of signals for offline analysis.

Contact measurements, where the sensor–tissue separation is 0 mm, were achieved by covering the PPG sensor in a clear plastic sheath. Measurements with the use of the plastic sheath were considered as direct contact between the sensor and the buccal mucosal lining with the assumption of the sheath being of negligible thickness. The noncontact geometries were achieved using an adjustable distance apparatus, as shown in Fig. 2(b). The apparatus utilizes a customized clean, clear rectal catheter (Pennine Healthcare, Derby, United Kingdom), with inner dimensions of 13.2 mm and a length of 40 mm. To adjust the distance within the catheter, four catheter caps of fixed positions were designed and printed. The fixed distances corresponded to sensor–tissue separations of 2.3 (i.e., catheter wall thickness), 5, 8, and 10 mm. The details of the sensor design have been discussed in an earlier publication.^17^

#### 2.2.2 Volunteer study and signal analysis

With institutional research ethics approval, healthy volunteers were invited to participate in the study. Twenty healthy volunteers (10 female and 10 male) with ages ranging from 18 to 38 years (mean age 28 years with a standard deviation of 4 years) were recruited based on the completion of a patient questionnaire regarding current and past medical conditions. Following a full explanation of the study objective, informed consent was obtained from all subjects prior to the study. The *in vivo* study was carried out in the Research Centre for Biomedical Engineering Physiological Measurements Laboratory. The room temperature was maintained at an average temperature of 20°C and the volunteers were rested in a semi-Fowlers position. Buccal PPG measurements were obtained at five sensor–tissue separations, 0, 2.3, 5, 8, and 10 mm, where the PPG sensor, either in the clean plastic sheath or adjustable distance apparatus, was positioned in the maxillary vestibule, directed toward the left cheek buccal mucosa lining. All PPG measurements were acquired at two light intensities by driving the LEDs simultaneously with currents of 20 and 40 mA, respectively. Two minutes of recorded data at each distance were saved, where signals were filtered and the ac and dc components were extracted, the details of which have been discussed in our earlier publication.^29^ The overall study duration lasted for up to 45 min, where short breaks were provided between changes of the sensor–tissue separations and the driving currents of the LEDs. Before and after the study, all reusable materials were disinfected using sterilizing fluid (Milton, United Kingdom) and volunteers were given a chance to rinse their mouths with mouthwash.

## 3 Results

### 3.1 Dependence on Sensor–Tissue Separation

**Fig. 3:**
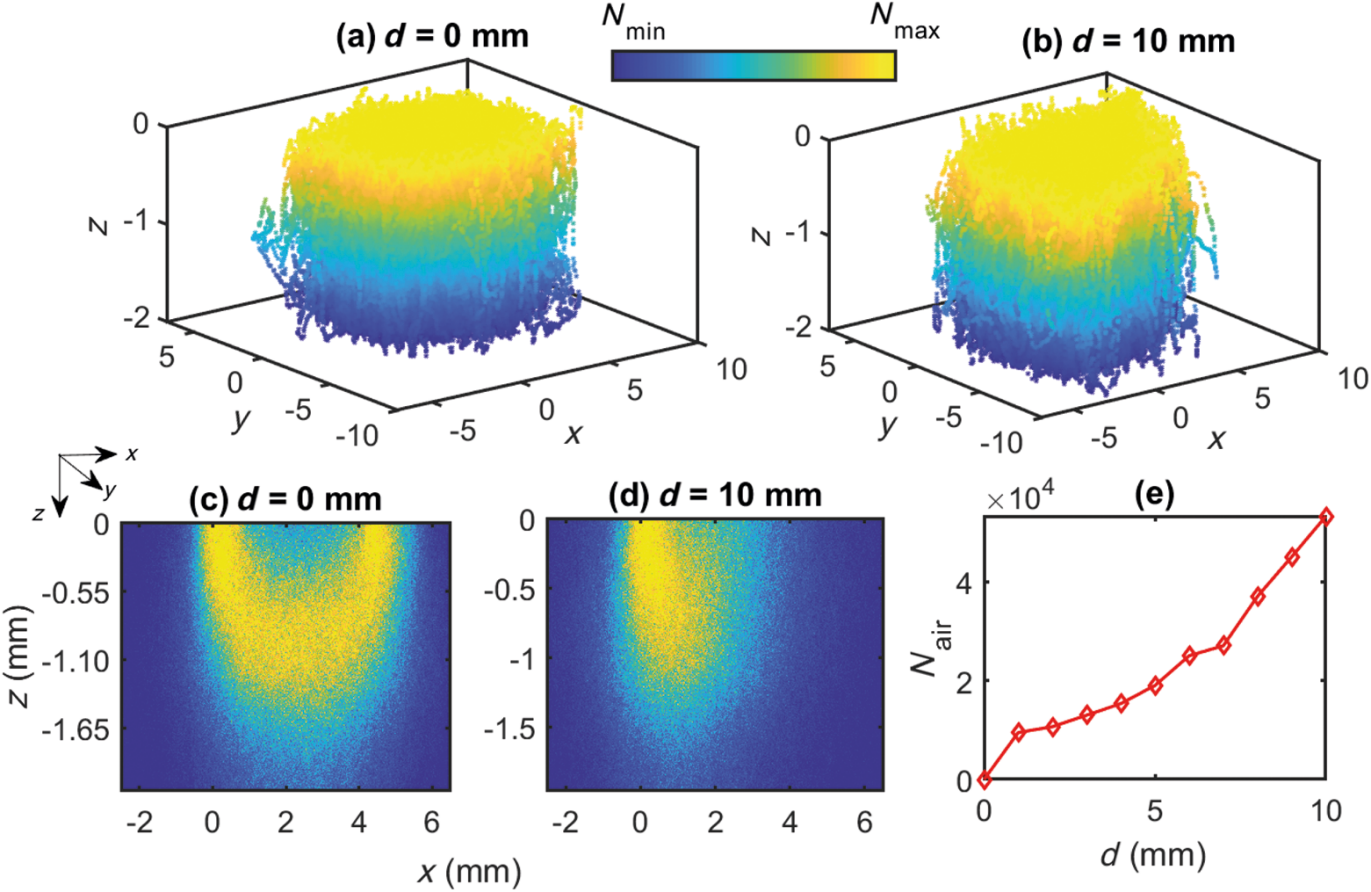
Simulated interactions between the optical radiation at 660 nm and the buccal tissue at separations of (a) 0 mm and (b) 10 mm are presented in 3-D Cartesian coordinate system xyz. The projections of the scatterplots at the xz plane are demonstrated in (c) and (d), respectively. Colorbar represents the distribution between the maximum and the minimum values, i.e., $N_{\max}$ and $N_{\min}$, respectively. (e) $N_{\text{air}}$ presents the number of photon-reflections at the air–tissue boundary before the photons reach the detector and is illustrated through the 0- to 10-mm sensor–tissue separations.

Monte Carlo simulated distribution of optical interactions at the wavelength of 660 nm at the contact and noncontact (separation distance = 10 mm) geometries are shown in Figs. 3(a) and 3(b), respectively. The 3-D scatterplots at the contact and noncontact geometries in Figs. 3(a) and 3(b) show accumulations of the maximum number of photon packets in the upper tissue layers followed by a reduction in the photon density through the depth of the tissue. Although the overall photon distribution does not show any significant differences between the contact and noncontact simulations, the projections of the scatterplots in the xz plane in Figs. 3(c) and 3(d) exhibit different patterns between them. The penetration depth in the contact-geometry is slightly higher compared to the noncontact geometry. In Fig. 3(c), the most probable path of photons takes a banana-like shape. On the contrary, as shown in Fig. 3(d), photons tend not to follow the familiar banana-shape. They also do not accumulate near the detector. Given that the sensor is located at a distance from the tissue, the photons exiting the tissue–surface experience multiple reflections at the air–tissue boundary before reaching the detector, causing noise in the detected signal. To further elaborate on this, the number of reflections in the air–tissue boundary ($N_{\text{air}}$) is shown in the Fig. 3(e) through the range of sensor–tissue separations (0 to 10 mm). Apparently, the number of reflections increases consistently with the sensor–tissue separations. This observation from the simulation agrees with our previously published experimental findings,^17^ which showed a higher noise level at the higher sensor–tissue separation.

**Fig. 4:**
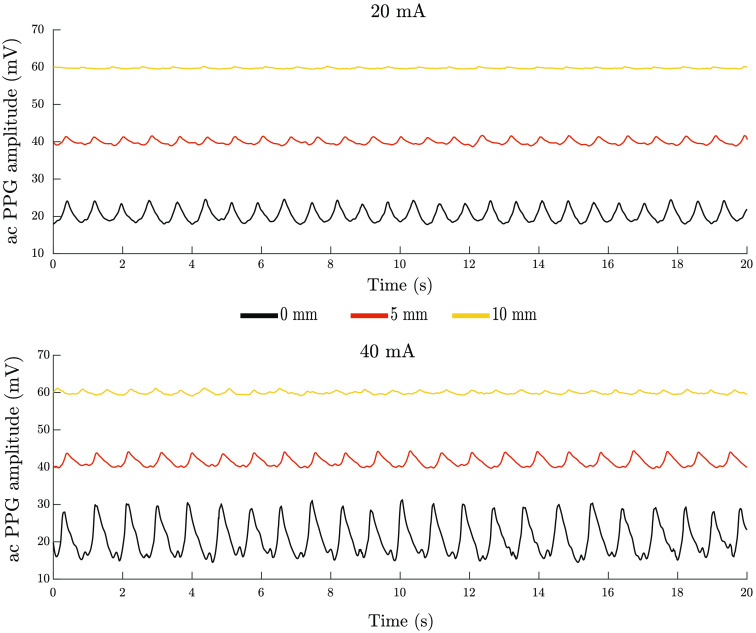
The ac amplitude of the PPG signal recorded from the buccal tissue of a healthy volunteer throughout 20 s at sensor–tissue separation distances 0, 5, and 10 mm at two LED driving currents, 20 and 40 mA, are presented in the top and bottom graphs, respectively.

The dependence of the measured PPG signals on the sensor–tissue separation is shown in Fig. 4. Results show the ac amplitudes extracted from the PPG signals recorded *in vivo* from the buccal tissue of a healthy volunteer with the sensor in contact with the tissue surface, and then increasing the sensor–tissue separation by 5 and 10 mm. A diminishing in the ac PPG amplitudes is noticed with the increasing sensor–tissue separation. Results with different LED-driving currents exhibit similar trends, but slightly higher amplitudes for the higher driving current.

**Fig. 5:**
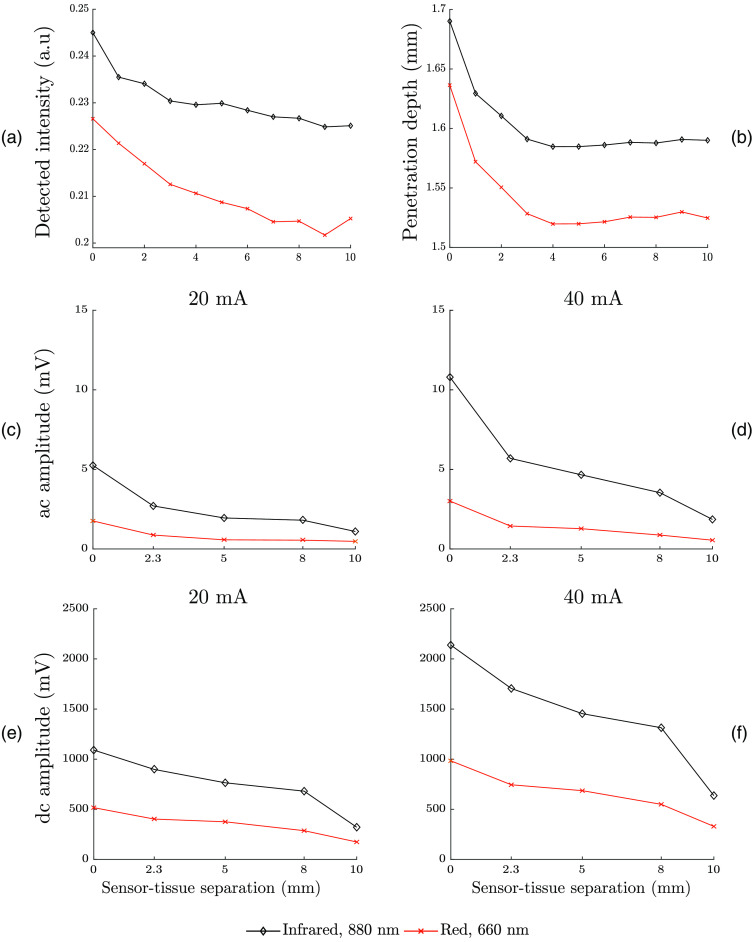
Simulated detected intensity and penetration depth through the buccal tissue volume at 660- and 880-nm optical wavelengths are presented in (a) and (b), respectively. PPG signals obtained experimentally were analyzed and the ac and dc amplitudes of the signals were extracted. The ac and dc amplitudes at the driving current 20 mA are presented in (c) and (e), respectively. The ac and dc amplitudes at the driving current of 40 mA are presented in (d) and (f), respectively.

**Fig. 6:**
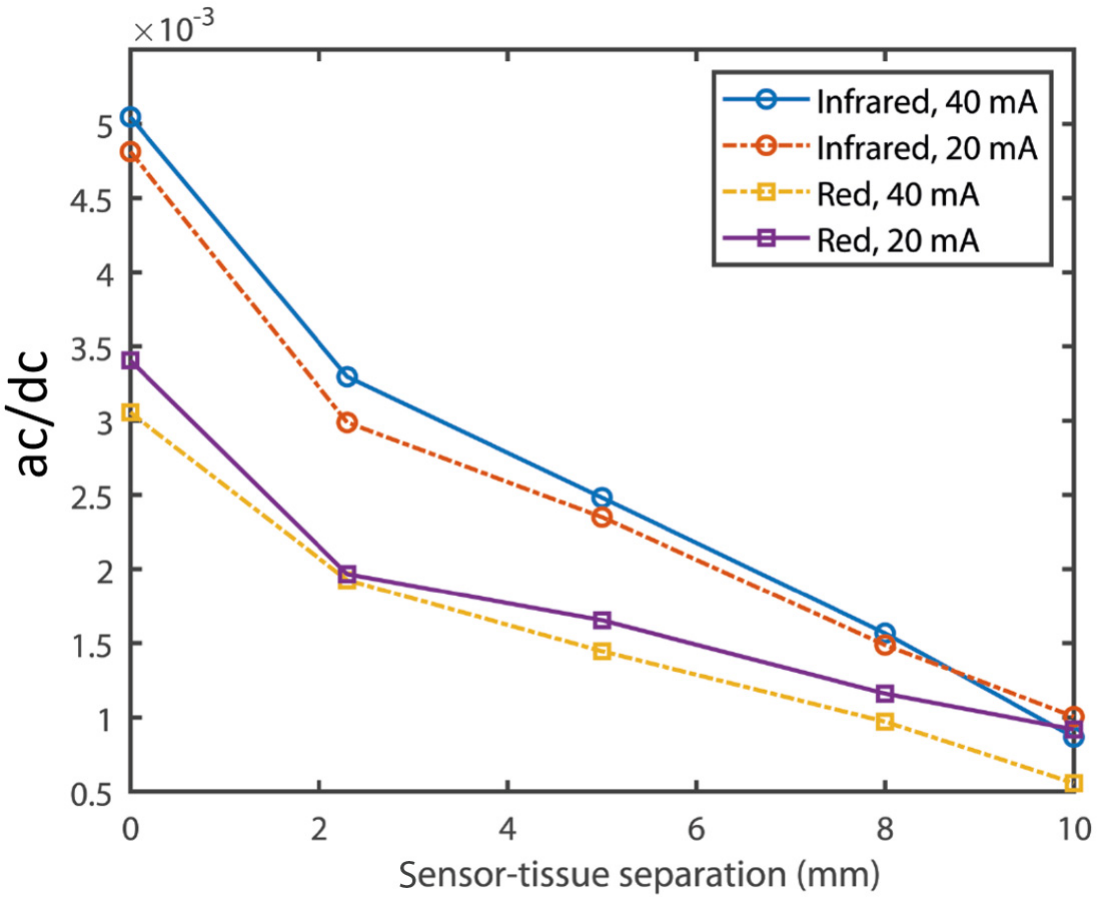
The ratios between the ac and dc PPG amplitudes at the red (660 nm) and the infrared (880 nm) for the driving currents 20 and 40 mA are presented along with sensor–tissue separations of 0 to 10 mm. Markers symbolize the data points.

The results from the detailed analysis with the simulated and experimental data are presented in Fig. 5. The simulation was carried out for a range of sensor–tissue separation from 0 to 10 mm with an interval of 1 mm. The experimental measurements were carried out at the sensor–tissue separations of 0, 2.3, 5, 8, and 10 mm. Both simulated and experimental results show higher infrared detected intensity compared to red. As shown in Fig. 5(a), the simulated intensities at red and infrared gradually decay with the increasing sensor–tissue separation. This observation was in agreement with experimental finding as shown in Figs. 5(c)–5(f) where both ac and dc amplitudes of the PPG signal at red and infrared wavelengths decrease gradually with the increasing sensor–tissue separation. The measurements with higher driving LED currents show the signals with similar trends and higher amplitudes. It should be noted that the simulated ‘intensity’ carries a similar meaning to the “amplitude” of the experimentally detected signals and should not be confused with the driving current intensity. Variation in depth of penetration with the sensor–tissue separation is presented in Fig. 5(b). Infrared light penetrates deeper compared to the red light. It is seen from the simulation that the penetration depth is the maximum when the sensor is in contact with the tissue surface, and it decays rapidly with the increasing separation and becomes almost constant after a separation distance of 5 mm. The ratio of the ac and dc PPG signals along the sensor–tissue separations for the two wavelengths with the different driving currents are shown in Fig. 6. The ratio decreases with the increasing sensor–tissue separation with the infrared ratios being higher compared to the red.

### 3.2 SNR and DIR

**Fig. 7:**
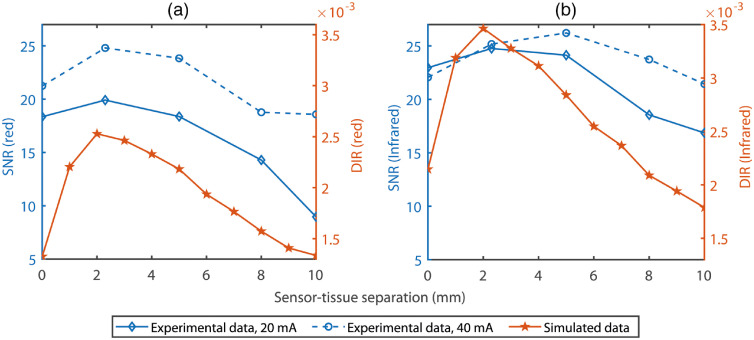
Demonstration of the SNR (experimental) and DIR (simulated) along with the sensor–tissue separations. SNR and DIR at red (660 nm) and infrared (880 nm) optical wavelengths are presented in (a) and (b), respectively. Results for the driving currents of 20 and 40 mA are presented by the solid and dashed blue lines, respectively. The red solid line represents the simulated data. SNR and DIR are presented along the left and right y axes, respectively. For better comparison, the axes have been limited to the same scales.

Figure 7 shows the comparison between the DIR determined from the simulated results and the signal-to-noise ratio (SNR) calculated from the experimental data. SNRs for the PPG signals acquired using 20 and 40 mA driving currents at 660 and 880 nm, along with the simulated DIRs, as functions of the sensor–tissue separations, are plotted in Figs. 7(a) and 7(b), respectively. Similar patterns are exhibited between the simulated and experimental results. The correlations between the simulated and experimental results were quantified by calculating the Pearson product moment correlation coefficients (R) using the four sets of data: (1) SNR at 660 nm measured with 20 mA driving current and DIR simulated at 660 nm, (2) SNR at 660 nm measured with 40 mA driving current and DIR simulated at 660 nm, (3) SNR at 880 nm measured with 20 mA driving current and DIR simulated at 880 nm, and (4) SNR at 880 nm measured with 40 mA driving current and DIR simulated at 880 nm. As presented in Table 2, all four sets show positive correlations. A moderately strong positive correlation (R>0.6) is found in the first set of data, whereas very strong positive correlations (R>0.8) are found in the other three sets of data.

**Table 2:** Pearson product-moment correlation coefficients (R) between the experimentally assessed SNR and the simulated DIR for the four sets of acquired signals.

Signal	SNR–DIR correlation coefficient (R)
λ=660 nm at 20 mA operating current	0.6553
λ=660 nm at 40 mA operating current	0.8765
λ=880 nm at 20 mA operating current	0.8141
λ=880 nm at 40 mA operating current	0.8381

### 3.3 Dependence on Tissue Perfusion and Oxygen Saturation

**Fig. 8:**
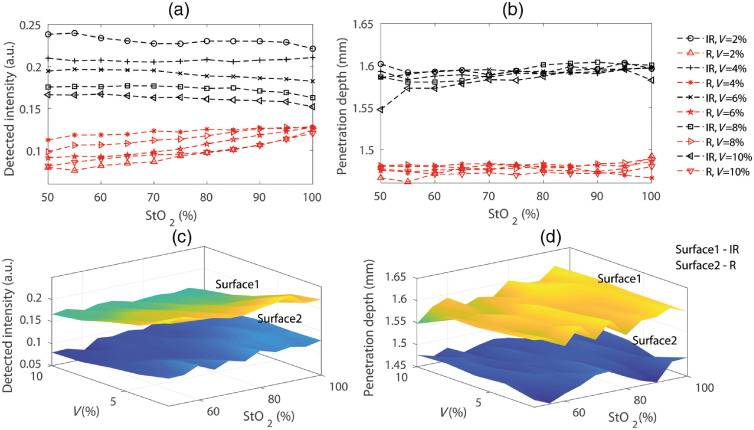
Simulation results for the detected intensities and the penetration depths at the variable states of buccal perfusion and oxygen saturation are presented. Results presented as two-dimensional line plots in (a) and (b) which have been illustrated separately as 3-D surface plots in (c) and (d), respectively, for clearer visualization.

Variable states of buccal perfusion and oxygen saturation were modeled *in silico* by manipulating the parameters to evaluate the submucosal absorption coefficient in Eq. (4). The simulated intensities and penetration depths at red and infrared optical wavelengths through the buccal tissue volume for a range of arterial oxygen saturation (${StO}_{2}=50\text{–}100\%$) and blood volume (V=2–10%) are presented in Figs. 8(a) and 8(b). For clearer visualization, the same results are presented in surface plots in Figs. 8(c) and 8(d), respectively. Simulations were carried out keeping the sensor and tissue surface in contact. As seen in the diagram, infrared intensity and penetration depths are higher compared to red in all blood volumes and oxygen saturations. With increasing blood volume, the intensity decays consistently at both the wavelengths. With increasing blood oxygen saturations, infrared intensity does not exhibit any significant change whereas red intensity is seen to increase slowly. The increase rate is higher for higher blood volume. The penetration depth does not seem to change significantly with the changes in blood volume and oxygen saturation. The infrared photons tend to be confined within a penetration depth of 1.55 to 1.6 mm, whereas the red photons tend to be confined within a depth of 1.45 to 1.47 mm.

**Fig. 9:**
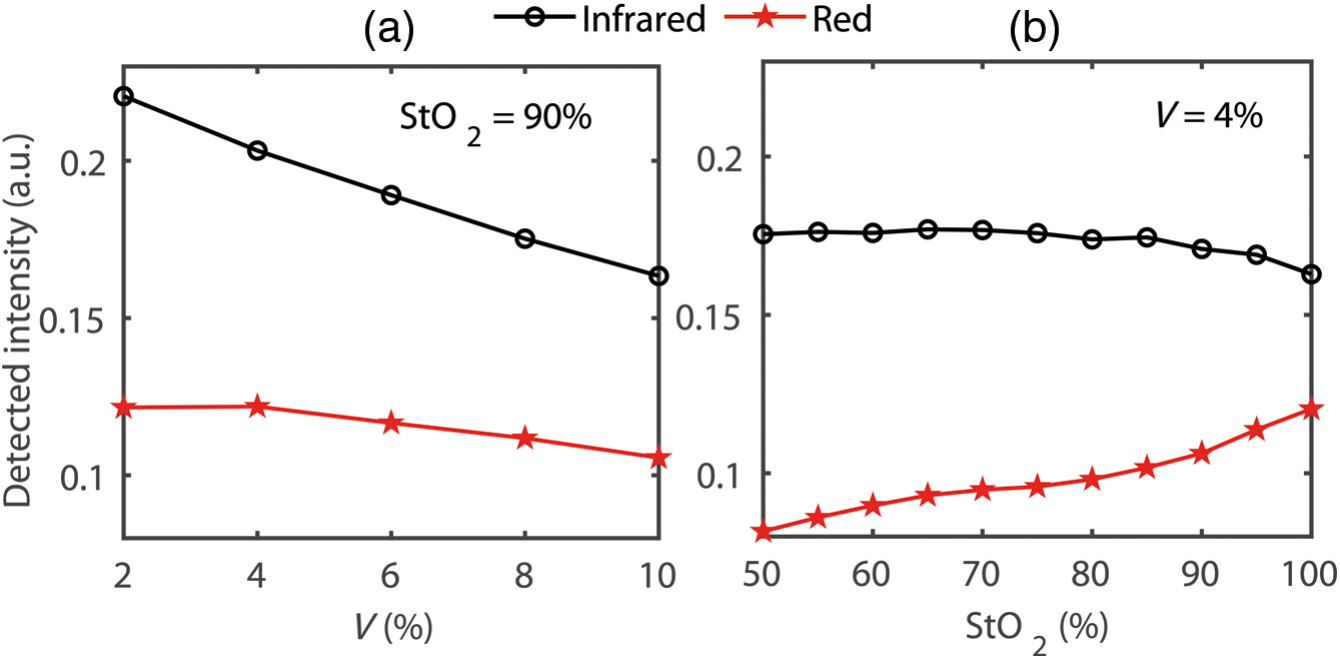
Variation in the simulated detected intensity: (a) with the changes in blood volume at a typical oxygen saturation of 95% and (b) with the tissue oxygen saturation at a typical blood volume of 4%.

The variation in the “detected intensity” with the blood volume and oxygen saturation is further elaborated in Fig. 9. Simulated intensity as a function of the buccal submucosal blood volume at a typical oxygen saturation ${\mathrm{StO}}_{2}=90\%$ is shown in Fig. 9(a). On the other hand, the simulated intensity as a function of the buccal submucosal total blood oxygen saturation at a typical blood volume V=4% is presented in Fig. 9(b). A consistent decay in the simulated intensity is apparent with the increased blood volume, and the changes are more prominent in the infrared light compared to red. With the increased oxygen saturation, however, red and infrared lights exhibit different patterns: infrared intensity slowly decreases whereas the red intensity gradually increases with the increased oxygen saturation.

## 4 Discussions

A surrogate-based *in silico* and *in vivo* characterization of an endocavitary PPG sensing device has been presented. Buccal tissue layer has been modeled as a proxy tissue-site to the colon because of its ease of access and morphological similarity. Though the tissue volumes differ structurally, they were considered optically similar based on the fact that the overall dimensions of the tissue volumes are much larger than the spatial distribution of photons.

Monte Carlo method has been chosen for the simulation as it provides several important advantages over other approaches (e.g., diffusion approximation, random walk model, etc.) such as^30^—(a) flexibility regarding the size, shape, and position of the optical source and detector, (b) inclusion of any level of complexity and heterogeneity in the tissue structure, (c) incorporating all physical processes between light and tissue such as multiple scattering, scattering anisotropy, high absorption, reflection, refraction, etc., and (d) ability to produce accurate results. The accuracy of the method can be quantified by its convergence rate, given by $1/\sqrt{Q}$, where Q is the number of simulations.^31^ The total number of iterations in each simulation (including the detected, discarded, and lost photons) was $Q≃10^{8}$, resulting in a convergence rate of 0.0001. A high number of iterations produced a reliable and accurate result in this work. For simulating the noncontact PPG sensing, the air medium was considered to have negligible attenuation, and the step sizes were calculated randomly (utilizing the pseudorandom numbers generated by MATLAB).

The proposed sensor has shown its potential to detect the PPG signals in both contact and noncontact geometries. Signal strength, which was equivalent to the “detected intensity” (simulated) and “ac PPG amplitude” (experimental), was compromised at higher noncontact separations. The simulated and experimental results confirmed that a good quality signal can be achieved within a sensor–tissue separation of 5 mm.

Simulated penetration depth decreased with increasing sensor–tissue separations. The minimum depth penetrated by light from red and infrared LEDs were 1.5 and 1.6 mm, respectively, within the deepest layer of the tissue volume (i.e., muscularis). We infer from this observation that the designed sensor is capable of collecting information from the deepest tissue layer from a distance as far as 1 cm from the tissue surface.

To simulate the changes in perfusion states of buccal tissue, the blood volume and oxygen saturation in the submucosal layer were varied because of the densest vasculature present in this tissue layer both in buccal and colon tissue. The ranges of blood volume (2% to 10%) and oxygen saturation (50% to 100%) were chosen based on the experimental findings reported previously.^23^

For every set of simulated physiological states (i.e., various combinations of blood volume and blood oxygen saturation of buccal submucosa), infrared intensities were higher compared to red. The infrared intensity was lower for higher blood volume and almost constant for higher oxygen saturation. With higher blood volume, the red intensity was also lower, however, with higher oxygen saturation, red intensity increased. This observation can be explained with the absorption properties of the two simulated blood absorbers, i.e., oxyhemoglobin and deoxyhemoglobin. With an increase of oxygen saturation, the optical properties of oxyhemoglobin prevail, absorbing more light at 880 nm compared to 660 nm. This leads to lesser attenuation of red light at higher oxygen saturation, especially in higher blood volumes, resulting in a gradual increase in the detected optical signal.

The muscularis of buccal tissue (which is similar to the colon muscularis) scatters more light in red compared to infrared.^22^ Due to the changes in the effective absorption coefficient, infrared light penetrated slightly deeper at higher oxygen saturation; however, this effect was negligible in the case of red light, leading to almost constant penetration depth.

DIR, introduced for the first time in this paper, is a crucial parameter that quantified the attenuation of the photon. DIR was found to increase initially up to d=2  mm and then to fall gradually with the increased sensor–tissue separation. This feature can be explained by the contribution of two attenuating factors: (a) absorbance in the medium and (b) reflection in the air–tissue boundary. In the contact or semicontact geometries (d≤2  mm), DIR was predominantly affected by the absorbance, whereas for higher noncontact separations, the effect of the air–tissue reflections prevailed. When the sensor was placed in contact or very close to the tissue–surface, light penetrated deeper compared to the higher noncontact separations. As light traveled further, photons interacted more with the tissue resulting in higher absorption in the tissue-layers and annihilation if they reached the threshold. Thus, in order to detect the same number of photons ($N_{\det}$), more incident photons ($N_{\mathrm{inc}}$) were simulated at the contact or semicontact geometry. Recalling Eq. (6), the higher $N_{\mathrm{inc}}$, the lower DIR. For example, at a semicontact sensor–tissue separation d=1  mm, to detect an $N_{\det}=10^{8}\text{ photons}$, an $N_{\mathrm{inc}}=8\times 10^{10}$ number of incident photons were required, which was twice that required to detect the same photons at a higher noncontact separation d=6  mm, which is $N_{\mathrm{inc}}=4\times 10^{10}$, resulting in a lower DIR at 1 mm compared to a 6-mm separation [i.e., DIR (1 mm) = 0.001 and DIR (6 mm) = 0.002]. As shown in the simulation, the penetration depth remained almost constant at d≥3  mm. In this region, the variation in DIR was caused due to the multiple reflections in the air–tissue boundary. Predominantly in this region, the simulated DIR and the experimental SNR exhibited strong qualitative and quantitative positive correlations. The photons reaching the detector perimeter reflected multiple times ($N_{\text{air}}$) at the air–tissue boundary, and $N_{\text{air}}$ increased with the increasing sensor–tissue separation. The multiple reflections before the detection of the photon physically resulted in the “noise” in the signal, explaining the noisy signals acquired experimentally at higher noncontact separations.^17^ Both the simulated and experimental data confirmed that good signals can be achieved using the sensor within a separation of 5 mm.

## 5 Conclusion

The feasibility of a dual-wavelength PPG sensor for intestinal viability monitoring has been evaluated through a set of simulations and experiments using a proxy tissue site. Results suggest the ability to obtain good quality PPG signals at different states of tissue perfusion and oxygenation. Good quality signals are expected through the range of 5-mm sensor–tissue separation distance. Following the satisfactory evaluation presented in this paper, a clinical proof of concept study is being planned to test the intestinal optical sensor as a tool to monitor intestinal viability during and after surgery.
